# Replication of Staphylococcal Resistance Plasmids

**DOI:** 10.3389/fmicb.2017.02279

**Published:** 2017-11-23

**Authors:** Stephen M. Kwong, Joshua P. Ramsay, Slade O. Jensen, Neville Firth

**Affiliations:** ^1^School of Life and Environmental Sciences, University of Sydney, Sydney, NSW, Australia; ^2^School of Biomedical Sciences, Curtin University, Perth, WA, Australia; ^3^Antimicrobial Resistance and Mobile Elements Group, Ingham Institute for Applied Medical Research, Sydney, NSW, Australia

**Keywords:** staphylococci, multiresistance plasmid, plasmid replication, replication initiation protein, plasmid copy number control, antisense RNA

## Abstract

The currently widespread and increasing prevalence of resistant bacterial pathogens is a significant medical problem. In clinical strains of staphylococci, the genetic determinants that confer resistance to antimicrobial agents are often located on mobile elements, such as plasmids. Many of these resistance plasmids are capable of horizontal transmission to other bacteria in their surroundings, allowing extraordinarily rapid adaptation of bacterial populations. Once the resistance plasmids have been spread, they are often perpetually maintained in the new host, even in the absence of selective pressure. Plasmid persistence is accomplished by plasmid-encoded genetic systems that ensure efficient replication and segregational stability during cell division. Staphylococcal plasmids utilize proteins of evolutionarily diverse families to initiate replication from the plasmid origin of replication. Several distinctive plasmid copy number control mechanisms have been studied in detail and these appear conserved within plasmid classes. The initiators utilize various strategies and serve a multifunctional role in (i) recognition and processing of the cognate replication origin to an initiation active form and (ii) recruitment of host-encoded replication proteins that facilitate replisome assembly. Understanding the detailed molecular mechanisms that underpin plasmid replication may lead to novel approaches that could be used to reverse or slow the development of resistance.

## Introduction

Plasmids are accessory extra-chromosomal genetic elements that provide bacteria with various adaptive qualities that have contributed to their success in diverse environmental niches. Over the last seven decades, the use of antimicrobial compounds in medical, veterinary, and agricultural practices has provided strong evolutionary selection for the acquisition of pre-existing and newly evolved antimicrobial resistance genes for bacterial survival. Plasmids have been instrumental in the dissemination of these resistance genes, and the rapid evolution of multiply resistant strains of *Staphylococcus aureus* in hospitals throughout the world provides an exemplar of this process.

*Staphylococcus aureus* is commonly normal flora of healthy individuals but is capable of causing serious life-threatening conditions, predominantly in debilitated individuals or patients undergoing surgical procedures. They have long been a primary cause of nosocomial infections and are notorious for the propensity to develop resistance to multiple antimicrobial agents. Particularly concerning has been the emergence of multidrug-resistant community-associated strains of *S. aureus* capable of causing highly virulent infections in healthy populations (Nimmo, 2012; Planet, 2017). In staphylococci, resistance genes are primarily associated with mobile genetic elements such as plasmids, genomic islands, and transposons (Lyon and Skurray, 1987; Schwarz et al., 2014). Many of these elements are capable of horizontal transfer between bacterial cells by conjugation, mobilization, and/or phage-mediated mechanisms, thus promoting the spread of resistance genes. In *S. aureus*, conjugative plasmids play a central role in enabling intercellular DNA transfer of both conjugative and mobilizable plasmids, which are each capable of accruing multiple resistance and/or virulence genes. It has recently been demonstrated that many of the large staphylococcal multiresistance plasmids, that were previously thought to be non-mobilizable, can in fact be mobilized through the carriage of *oriT* mimic sequences (O’Brien et al., 2015; Ramsay et al., 2016; Ramsay and Firth, 2017). The transfer of these types of plasmids can facilitate multidrug resistance evolution in a single step. Thus, both conjugative and mobilizable plasmid classes are important adaptive tools that have had a major impact on the evolution of antimicrobial resistance.

In the event of plasmid horizontal transfer, a single-stranded copy of the plasmid is transmitted from the donor cell to a recipient cell where it is re-circularized and replicated into a double-stranded form. If the transferred plasmid is capable of efficient autonomous replication in the recipient and becomes established, the plasmid and its resistance genes are rarely lost. Most small plasmids counteract loss during cell division by replicating at high copy numbers. In this situation, there are many plasmid copies that are randomly distributed to daughter cells as the cytoplasmic contents of the parent cell are shared. It is uneconomical for larger plasmids to use this strategy due to the metabolic and genetic loads imposed upon the host cell that would encumber fitness. Large plasmids instead encode various segregational stability systems, often including active partitioning, post-segregational killing, and multimer resolution, that work together to maintain extremely high inherent stability, enabling them to replicate with low copy numbers, presumably to minimize the burden on the host. Crucial to plasmid survival, in terms of both segregational stability and fitness cost, is replication, which can be initiated when needed during cell division and turned off once the ideal plasmid copy number is established after division. This essential capacity to sense and adjust plasmid quantity leads to a defined average copy number in any given host. Plasmids display considerable diversity in their replication systems, the components used to initiate replication and the mechanisms by which replication is controlled (del Solar et al., 1998; Brantl, 2014). Comprehensive reviews on the replication of circular bacterial plasmids (del Solar et al., 1998) and of rolling-circle plasmids (Khan, 1997; Ruiz-Masó et al., 2015) are available in the literature. This article reviews our current understanding of the replication initiation mechanisms of staphylococcal plasmids (last reviewed by Novick, 1989), the systems they use to control plasmid copy number, and includes an updated view of the distribution of *S. aureus* plasmid types.

## Classification of Staphylococcal Plasmids

Staphylococcal plasmids range from just over 1 kb to greater than 60 kb in size. In general, the smaller plasmids (between 1 and 8 kb) are cryptic or encode a single resistance determinant and replicate via a rolling-circle replication (RCR) mechanism that is hallmarked by the production of single-stranded intermediates during replication. Many similarities can be drawn between the replication of RCR plasmids and some classes of bacteriophages (e.g., ΦX174), with the major difference being the control of replication frequency. Bacteriophage often propagates their genomes in bursts without consideration to the host’s survival, whereas plasmids are host-dependent and thus control their replication to be synergetic with that of the host. Staphylococcal RCR plasmids (also known as class I staphylococcal plasmids) can be further sub-grouped into plasmid families based on the evolutionary relationships of their essential replication initiation proteins. Four main subclasses of RCR plasmids have been found in staphylococci and are represented by the prototypes pT181, pC194, pE194, and pSN2, which are distinguished by the type of replication initiation gene that they carry (Novick, 1989; Firth and Skurray, 2006). Some RCR plasmids have been extensively studied including closely related tetracycline resistance plasmid pT181 and chloramphenicol resistance plasmid pC221 of the pT181 family (Thomas et al., 1995; Khan, 1997). Characterized members of the pC194 family include pC194 itself, conferring resistance to chloramphenicol (Gruss et al., 1987), and the aminoglycoside resistance plasmid pUB110 (Maciag et al., 1988). The best studied plasmid of the pE194 family is tetracycline resistance plasmid pMV158, originally isolated from streptococci but shown to be capable of stable replication in a wide range of bacterial species (Meijer et al., 1995). pSN2 family members have been studied to a lesser extent in regard to their replication and control mechanisms.

Staphylococcal plasmids greater than 8 kb in size typically utilize a theta (𝜃)-type replication mechanism and have historically been divided into two main classes depending upon their conjugative ability (Novick, 1989; Firth and Skurray, 2006). Non-conjugative theta-replicating plasmids include the well-known β-lactamase/heavy-metal resistance plasmids, pSK1-family multiresistance plasmids, and pSK639-family plasmids (Firth and Skurray, 2006). The conjugative plasmids have traditionally included closely related multiresistance plasmids exemplified by pSK41 (Liu et al., 2013) and pGO1 (Caryl and O’Neill, 2009); however, two new distinct families have recently been described (Ramsay et al., 2016). In general, the conjugative plasmids are larger than the non-conjugative plasmids due to the carriage of extensive gene arrays that encode a type IV secretion system (T4SS), a large multiprotein pore complex through which single-stranded DNA can be transferred to the recipient cell, a nicking relaxase enzyme and its DNA substrate (*oriT*), relaxase accessory proteins, and a coupling protein that provides the basis of recognition between the relaxase and the mating pore. RCR plasmids of the pT181, pC194, and pE194 families and some members of the theta-replicating pSK639-family are known to carry only the relaxase unit (*pre* or *mob* genes and *oriT*), which enables horizontal transfer via mobilization. Plasmid mobilization can occur if these plasmids are able to exploit the mating pore provided by a suitable co-resident conjugative plasmid (or other conjugative element).

## Plasmid Incompatibility

Non-identical plasmids that share nearly identical replication/maintenance components (DNA, RNA, and/or proteins) display plasmid incompatibility. That is, they are unable to be maintained efficiently through continued rounds of cell division in the absence of plasmid selection. Incompatibility is caused by the inability of the *trans*-acting replication or maintenance components to distinguish “self” from “non-self,” and has historically been used as an indication of plasmid relatedness. Staphylococcal plasmids have been placed into at least 15 incompatibility groups (Ruby and Novick, 1975; Iordanescu and Surdeanu, 1980; Novick, 1989; Udo and Grubb, 1991). Ten groups corresponded to RCR plasmids with the remainder being larger, and hence, probably theta-replicating. It was noted earlier that very closely related RCR plasmids were in different incompatibility groups and quite dissimilar theta-replicating plasmids were often in the same incompatibility group (Novick, 1989). This indicated that while incompatibility tests can yield biologically relevant information regarding the ability of plasmids to coexist stably, they do not necessarily indicate relatedness on a whole, particularly in regard to their resistance or other phenotypes. The recombinatory systems (described below) that have shaped the evolution of both RCR and theta-replicating plasmids provide an explanation for this phenomenon.

## Evolution of Resistance Plasmids

As the nucleotide sequences of RCR plasmids became available, it was apparent that they were composed of interchangeable modules or gene cassettes (Gruss and Ehrlich, 1989; Novick, 1989). Genetic features, such as resistance genes, mobilization systems, and lagging-strand replication origins were not conserved among plasmids of the same incompatibility group or replicon type. The gene cassette junctions were noted to be abrupt with the level of sequence identity dropping from near perfect to no homology across a single pair of nucleotides (Gruss and Ehrlich, 1989; Novick, 1989). The production of ssDNA in the RCR mechanism is critical to this type of cassette exchange due to the greatly increased capacity for homologous and illegitimate recombination events (Niaudet et al., 1984; Jannière and Ehrlich, 1987). Thus, a cassette can insert or replace another cassette if the appropriate flanking sequences are present in the target plasmid. An example of cassette dissemination by this mechanism is evident with the multidrug resistance locus *qacC* in members of the pC194 family where the gene is located between conserved elements required for leading and lagging strand replication (Leelaporn et al., 1995; Wassenaar et al., 2016). The composition and arrangement of gene cassettes and the associated flanking regions are shuffled by occurrences such as plasmid co-integration and aberrant replication events to generate new combinations (Ballester et al., 1989; Gruss and Ehrlich, 1989).

pT181 was discovered to have defined recombination sites, termed RS_A_ and RS_B_, that promote the formation of plasmid co-integrates by site-specific recombination (Novick et al., 1984). RS_B_ is a conserved sequence found in the pT181 lagging strand replication origin *palA* (aka *ssoA*), which is broadly carried by diverse staphylococcal RCR plasmids (Novick et al., 1989). Plasmids bearing lagging stand replication origin types *ssoU, ssoT*, and *ssoW* also possess the conserved RS_B_ sequence (Kramer et al., 1999). RS_A_ is found in the region upstream of *pre*, the protein product (Pre) of which is essential for recombination at this site (Gennaro et al., 1987). Later it was discovered that Pre is a member of the pMV158 Mob protein family, which was essential for the mobilization of this plasmid (Priebe and Lacks, 1989). Therefore, it is now understood that the primary role of pT181 Pre is in mobilization and its role in recombination is a by-product of its nicking activity at RS_A_, which functions as the pT181 *oriT*. Pre-mediated nicking of RS_A_ generates an efficient substrate for co-integrate formation between two plasmids (Priebe and Lacks, 1989).

Theta-replicating plasmids are known to carry transposons but often also contain one or more assemblies of resistance gene clusters inserted into a conserved plasmid backbone (Firth and Skurray, 2006). These regions often correspond to IS*257*-flanked co-integrated copies of small plasmids. The genetic arrangement of clusters resembles that of composite transposons but probably have not inserted as such. Rather the clusters are likely the result of IS*257* transposition by a non-resolved replicative mechanism leading to co-integration of two plasmids and resulting in directly repeated IS*257* copies at each junction (Needham et al., 1995; Leelaporn et al., 1996; Firth and Skurray, 1998). Often the resulting co-integrant is then fine tuned by intra-molecular transposition of IS*257*, which promotes sequence deletions of flanking DNA (e.g., the replication region of co-integrated RCR plasmids; Berg et al., 1998), and sequences within the terminal inverted repeats of IS*257* can modulate the expression of adjoining genes by generating hybrid promoters (Leelaporn et al., 1994; Simpson et al., 2000; Pérez-Roth et al., 2010). Thus, theta-replicating plasmids accrue resistance genes through the incorporation of smaller resistance plasmids and subsequent deletion of problematic or unnecessary sequences (Firth and Skurray, 1998). In addition to influencing the evolution of theta-replicating multiresistance plasmids, IS*257*-mediated RCR plasmid integration events also appear to have played a role in the evolution of staphylococcal genomic islands, such as SCC*mec* (Stewart et al., 1994; Firth and Skurray, 2006).

## Plasmid Replication Regions

**FIGURE 1:**
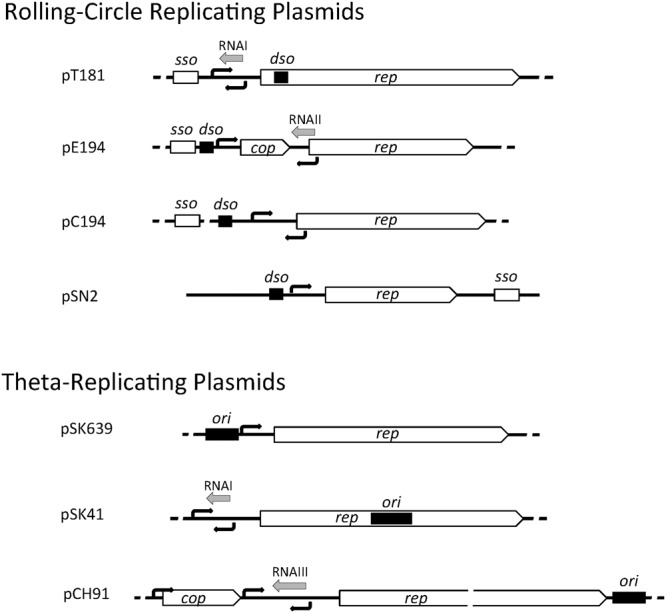
Genetic organization of staphylococcal plasmid replication regions. A representative of each of the seven plasmid initiator types are illustrated and include the *rep* gene for the replication initiation protein, *cop* gene encoding a *rep* repressor, origin of replication (*dso* or *ori*, black rectangles), predicted or determined promoters (black arrows), antisense RNAs (gray arrows), and single-stranded origin of replication (*sso*).

Staphylococcal plasmids carry a 1- to 2-kb region that contains genetic information for autonomous replication and its control. The essential components include (i) an origin of replication (*dso* in RCR plasmids or *ori* in theta-replicating plasmids), (ii) a replication control element (antisense RNA and/or protein), and (iii) a gene encoding the replication initiation protein, Rep. In RCR plasmids, the double-stranded origin, *dso*, contains a sequence-specific binding site for the Rep protein and a short, partially palindromic sequence. In pT181, Rep binding to the *dso* alters the conformation of this palindromic sequence to a cruciform structure that is efficiently nicked by Rep (Koepsel and Khan, 1986; Noirot et al., 1990). In most RCR plasmids, the Rep binding site and the nick site are adjacent, and in other plasmids (e.g., pMV158), the two sites are separated by a short distance of up to 100 bp. The *dso* is often positioned upstream of the *rep* coding region, except in pT181 family plasmids, where the *dso* is found within the *rep* coding region (**Figure 1**). In RCR plasmids, an additional element, the single-stranded origin, *sso*, is required for efficient lagging strand synthesis using the leading strand as template. Therefore, the *sso* is only functional on the leading strand and its orientation cannot be reversed (Gruss et al., 1987). The *sso* displays more variety in its position relative to the *rep* gene (**Figure 1**) and is often separated from it by various gene cassettes indicating that its position is flexible. *sso* sequences are known to limit the host range of RCR plasmids and currently five classes are known to exist: *ssoA, ssoU, ssoT, ssoW*, and *ssoL* (Ruiz-Masó et al., 2015). The ∼160 nt *ssoA* carried by pT181 family plasmids restricts their stable replication to staphylococcal species (Gruss et al., 1987) and *ssoA* sequences carried by other plasmid families also appear host-restricted (Kramer et al., 1998). In a single-stranded state, the *ssoA* is capable of forming a large secondary structure that is recognized and primed by RNA polymerase (RNAP) (Kramer et al., 1997). In addition to DNA polymerase III (PolC), DNA polymerase I (PolA) is required during elongation and termination of lagging strand synthesis (Diaz et al., 1994; Kramer et al., 1997). Members of the pC194 and pE194 families have been shown to carry a widely recognized RNAP-dependent *ssoU* permitting them to replicate in all *firmicutes* tested (Boe et al., 1989; Kramer et al., 1995; Lorenzo-Díaz and Espinosa, 2009). *ssoW* of lactococcal plasmid pWVO1 appears to be capable of both RNAP-dependent priming and primosome-dependent priming via a *ssoW*-located primosome assembly site; however, lagging strand replication of this plasmid is restricted to lactococcal species (Seegers et al., 1995).

In theta-replicating plasmids, the position of the origin of replication (*ori*) is variable. In pSK41, the *ori* has been shown to be contained centrally within *rep*, whereas in pSK639 and pCH91 family members, the predicted location of *ori* is upstream and downstream of *rep*, respectively (**Figure 1**). Although *dso* and *ori* both contain one or more binding sites for the replication initiator, they are functionally quite different. Cleavage of the parental DNA strands does not occur during theta-replication and instead a short region of the *ori* (or immediately next to it) is melted upon Rep binding to form an open single-stranded initiation complex.

## Replication Initiators

**FIGURE 2:**
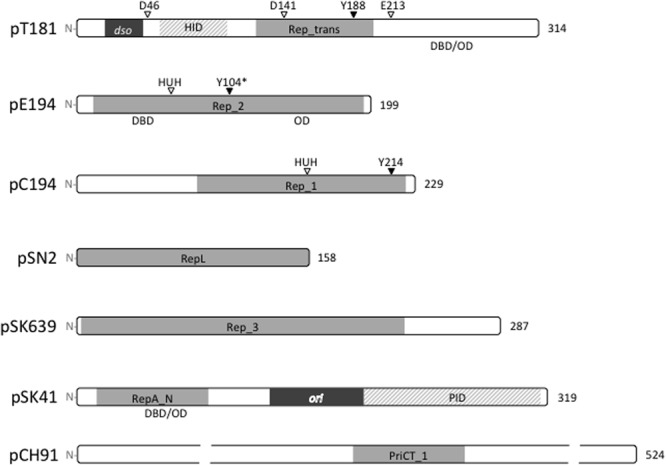
Replication initiation proteins of staphylococcal plasmids. The initiation proteins of prototypical plasmids are represented by bars that indicate the relative sizes of the proteins. Conserved domains within each plasmid replication initiator type are shaded gray. The HUH and DDE metal ion-binding motifs and catalytic tyrosine residues (Y) are labeled where known. ^∗^The Y104 of pE194 was predicted by generating an amino acid sequence alignment with the pMV158 RepB protein. In pT181 and pSK41 plasmid families, the origins of replication (*dso* and *ori*) are located within the *rep* coding regions and derived protein sequences in these regions are dispensable for replication. The pT181 RepC–PcrA interaction domain (HID) and the pSK41 Rep–DnaG primase interaction domain (PID) are indicated. Where known, the positions of DNA-binding domains (DBD) and oligomerization domains (OD) are also shown.

To date, staphylococcal plasmids have been found to encode one or more of seven distinct types of replication initiation protein, defined by the conserved domains Rep_trans (pfam02486; pT181), Rep_1 (pfam01446; pC194), Rep_2 (pfam01719; pE194), and RepL (pfam05732; pSN2) for the RCR plasmids, and Rep_3 (pfam01051; pSK639), RepA_N (pfam06970; pSK41), and PriCT_1 (pfam08708; pCH91) for the theta-replicating plasmids. The domain organizations for representatives of each protein family are illustrated in **Figure 2**. All of the initiators contain DNA-binding domains (DBD) for specific binding to their cognate *dso* or *ori*. In some cases, oligomerization domains (OD) have been identified. The RCR initiators are all expected to have topoisomerase activity essential for *dso* cleavage at initiation of replication and for cleavage and ligation during the termination of leading strand synthesis. Conserved catalytic tyrosine residues needed for these activities are also indicated where known (**Figure 2**).

**FIGURE 3:**
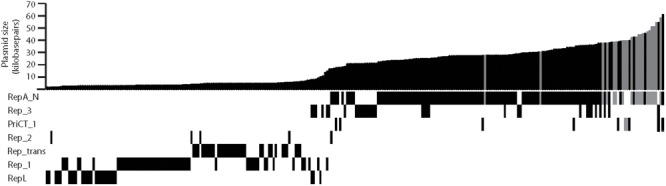
Distribution of replicon types among sequenced *Staphylococcus aureus* plasmids. The graph at the top indicates plasmid sizes for 278 plasmids in the databases. The conserved domains for each replication initiation protein are indicated by a black/gray bar below the graph. Some plasmids encode more than one Rep type. Plasmids predicted to be non-conjugative are represented by black bars and plasmids predicted to be conjugative are represented by gray bars.

The large number of completely sequenced staphylococcal plasmids now available has allowed us to present an updated view of the distribution of replication initiator genes that are typical of each plasmid family. Due to its clinical significance, plasmids from *S. aureus* clearly dominate the databases. It is currently not known whether distribution of the plasmid classes presented here would vary markedly in other species of the genus. Our analysis of the replication genes of 278 completely sequenced non-identical *S. aureus* plasmids is presented in **Figure 3** (Supplementary Data Sheet 1). Slightly more than half of the plasmids (57%) are predicted to replicate via a theta-type mechanism with the remainder using an RCR mechanism (**Figure 3**). As noted previously (Novick, 1989), the RCR plasmids appear restricted in size with > 90% less than 5 kb. Of the RCR plasmids, 45% were expected to utilize an initiator containing the Rep_1 conserved domain, 28% Rep_trans, 22% RepL, and only 3% Rep_2, with two novel plasmids that could not be grouped. Interestingly, there also appears to be a trend between RCR initiator type and plasmid size where RepL < Rep_1 < Rep_trans although there are also numerous exceptions to this trend (**Figure 3**).

Of 160 *S. aureus* theta-replicating plasmids, those using RepA_N initiators are clearly the most common, being encoded by approximately 80% (**Figure 3**). Rep_3 domain initiators are encoded by approximately 20% and PriCT_1 by about 6% of sequenced plasmids. A total of 11% of theta-replicating plasmids contained two different potential replication initiation genes of these types. In some cases, there is evidence that one of the replicons has been inactivated through genetic alterations (mutations/truncations). In plasmids with both *repA_N*- and *rep_3–*type genes, there are examples where either type appears inactivated. In other cases, there is no obvious defect in either replication region. In regard to the conjugative plasmids, use of RepA_N-type initiators is even more pronounced being used by 90% (18 out of 20), with only two cases of a PriCT_1-type replicon. Interestingly, there are no Rep_3-type plasmids that are predicted to be conjugative.

## Rolling-Circle Replication

### Rep_trans Plasmids – the pT181 Family

pT181 family plasmids are widespread in the staphylococci and frequently carry tetracycline or chloramphenicol resistance genes. The two best studied members include pT181 and pC221, and their respective initiators, RepC and RepD, share 82% overall sequence identity. Sequence identity (90%) increases across the common Rep_trans domains, which contain the essential catalytically active tyrosine residues (RepC Y188; RepD Y191). The major region of sequence divergence between the initiators lies in the C-terminal regions, which, for both proteins, has been shown to bind to their cognate *dso*s (Koepsel and Khan, 1986; Thomas et al., 1990). These variable regions have been shown to contain a six amino acid determinant that governs *dso*-binding specificity and limits cross reactivity between initiators and origins of different Rep_trans plasmids (Dempsey et al., 1992; Wang et al., 1992). As such, multiple incompatibility groups exist in the pT181 plasmid family potentially allowing them to stably coexist in the same host cell.

In pT181 family plasmids, the Rep protein binds as a dimer to the *dso* bending the DNA into a cruciform structure that exposes the nick site in its single-stranded form (Noirot et al., 1990). The topoisomerase activity of the initiator results in nicking at the *dso* and formation of a phosphodiester bond between the reactive tyrosine hydroxyl group and the newly created 5′ phosphate of the nicked strand (Thomas et al., 1990). The Rep protein also aids recruitment of PcrA helicase, which is essential to plasmid replication and unwinds and separates the leading parental strand from the lagging template strand (Slatter et al., 2009). Synthesis is initiated from the Rep-generated 3′ hydroxyl group and is known to require DNA polymerase III (Majumder and Novick, 1988). Synthesis of the leading strand continues full circle and 10–12 nt past the original nick site (Rasooly and Novick, 1993). The covalently attached Rep dimer then catalyses a second series of strand-transfer reactions that terminates leading strand replication, resulting in a double-stranded plasmid containing a newly synthesized leading strand, a re-circularized single-stranded displaced strand and a Rep dimer (RepC/RepC^∗^) that remains attached to the 10–12 nt adduct rendering the protein inactive for replication (Rasooly et al., 1994). This inactivation mechanism prevents reuse of the initiator for further rounds of replication and is a fundamental prerequisite for pT181 plasmid copy number control.

Crystallography and 3D modeling of Rep_trans initiators suggests that the dimeric structure resembles a horseshoe with a basic channel sitting atop the structure (containing the six amino acid specificity determinant) that could accommodate dsDNA (Carr et al., 2016). The catalytic residues, required for DNA nicking and ligation, including the Tyr active site, are positioned on the inner face. The divalent metal ion-binding site that is essential for topoisomerase activity is coordinated by three distally located residues DDE (**Figure 2**) brought together within antiparallel β-sheets (Carr et al., 2016). Residues affecting PcrA helicase interaction were mapped to the open end of the horseshoe (Carr et al., 2016).

### Rep_1 and Rep_2 Plasmids – the pC194 and pE194 Families

Although possessing distinguishable conserved domains, the Rep_1 and Rep_2 families of plasmid replication initiators are united by the HUH metal ion-binding motif and a catalytic Tyr residue which are both necessary for topoisomerase activity (Chandler et al., 2013). The HUH superfamily also includes bacteriophage replication proteins, conjugative DNA relaxases and transposases. Members of the HUH superfamily may have either one or two active tyrosine residues (Chandler et al., 2013). Plasmids in both families exhibit a broad host range with some members having been shown to replicate efficiently in a wide range of Gram-positive and Gram-negative bacteria and even eukaryotic cells (Weisblum et al., 1979; Goursot et al., 1982; del Solar et al., 1987; Coffey et al., 1994; Aleshin et al., 1999).

The pMV158 initiator RepB (210 residues) has been studied in detail and is one of only a small number of Rep proteins with structure determined by crystallography (Boer et al., 2009). Unlike the dimeric state of pT181 initiators, pMV158 RepB is instead shown to form a hexameric ring structure that may encircle the DNA and increase processivity of leading strand synthesis (Ruiz-Masó et al., 2004; Boer et al., 2009). The N-terminal region of RepB contains both the DNA binding and topoisomerase activities of the protein, while the C-terminal domain is required for hexameric oligomerization (Boer et al., 2016). The pMV158 *dso* contains a set of three directly repeated sequences (*bind*) located 84 bp downstream of the nick site, which is found in the loop of a palindromic secondary structure (Puyet et al., 1988). It has been shown that, at least *in vitro*, RepB can bind to DNA fragments containing either the nick site or the *bind* locus, although the affinity of the protein for the latter is much higher (Ruiz-Masó et al., 2007). On the other hand, RepB catalytic activity requires a single-stranded DNA substrate, and the requirement for plasmid supercoiling indicates that the nick site is presented in a single-stranded conformation only when in this topological state (del Solar et al., 1987; Moscoso et al., 1995). In contrast to the Rep_trans type initiators, the active Tyr residue in pMV158 RepB does not appear to form a stable covalent phosphodiester bond with the 5′-end of the cleaved strand (Moscoso et al., 1995). However, a more labile RepB-DNA covalent adduct was observed after rapid treatment of cleavage reactions with SDS and proteinase K (Ruiz-Masó et al., 2016), indicating that RepB inactivation after one round of leading strand synthesis might still occur through a similar mechanism.

The pC194 and pUB110 replication initiation proteins, RepA and RepU, are monomers in solution but bind to their *dso*s cooperatively in pairs (Noirot-Gros et al., 1994; Müller et al., 1995). At high concentrations, approximately six RepU monomers coat the *dso* region and this complex is able to extend upon the adjacent *repU* promoter leading to *repU* transcriptional silencing (Müller et al., 1995). The pC194 RepA protein has three catalytic residues, Tyr214, Glu142, and Glu210 that are essential for nicking and closing activities of the protein but that do not affect *dso* binding and these residues are universally conserved in all plasmids of the pC194 family (Noirot-Gros et al., 1994). The proposed roles of the essential residues are as follows: (i) nucleophilic attack at the nick site during initiation by Tyr214; (ii) hydrolysis of the regenerated nick site at the leading strand termination step promoted by Glu210 (which would act as a general base catalyst); and (iii) metal ion coordination involving Glu142 (Noirot-Gros et al., 1994). The equivalent residues in pUB110 RepU are Tyr241, Glu163, and Glu237 (Noirot-Gros et al., 1994; **Figure 3**). The 37 kDa RepU protein has two forms that show slightly different molecular weights in polyacrylamide gels and it has been suggested that the larger form could be attached to a short oligo (like pT181) or modified in another way that renders it inactive for replication (Müller et al., 1995). Although the precise mechanism used to prevent Rep recycling has not yet been elucidated, such a mechanism would be a prerequisite for effective copy number control (Gros et al., 1989). Interestingly, it has been reported that the Rep proteins of several RCR plasmids, including pC194, can also act as mobilization relaxases in the presence of the integrative conjugative element ICE*Bs1* (Lee et al., 2012).

### RepL Plasmids – the pSN2 Family

pSN2 family members are among the smallest RCR plasmids in staphylococci (**Figure 3**). Many pSN2 family plasmids are cryptic or carry a single resistance determinant, with erythromycin resistance being the most common. No members of this family are known to carry mobilization functions. The pSN2 *dso* and *ssoA* can be readily identified based on similarity to other RCR plasmids (Khan and Novick, 1982; Novick, 1989; Khan, 1997) and the *ssoA* was shown to be required for efficient lagging strand synthesis by an RNAP-dependent priming mechanism (Dempsey et al., 1995). Notably the pSN2 family RepL proteins contain a helix-turn-helix (HTH) DNA domain and are predicted to be about 18 kDa, whereas other RCR initiators are considerably larger (Novick, 1989; Catchpole and Dyke, 1992). At least two incompatibility groups have been shown to exist in the pSN2 family (Oliveira et al., 1993) with sequence variation most often occurring in the central part of the protein (residues 85–95), indicating the location of a possible DNA-binding specificity determinant. At this time, no copy number control mechanism has been identified although many pSN2 family members carry a short open reading frame (that is only sometimes annotated) encoding a small, basic protein (45–60 aa) that could potentially play a regulatory role.

## Theta-Mode Replication

### RepA_N Plasmids

Plasmid replication initiators containing the conserved RepA_N domain are frequently used by theta-replicating *S. aureus* multiresistance plasmids, both conjugative and non-conjugative, and are widespread in many other coagulase-negative staphylococcal species, including plasmids from *S. epidermidis, S. haemolyticus, S. saprophyticus, S. xylosis*, and *S. warneri*. The RepA_N-type initiators are also broadly distributed among other large plasmids of the low G+C Gram-positive firmicutes such as enterococci, lactobacilli, lactococci, and bacilli (Firth et al., 2000; Weaver et al., 2009).

The best studied RepA_N plasmid is the conjugative multiresistance plasmid pSK41, which confers resistance to the aminoglycosides kanamycin, tobramycin, gentamycin, and neomycin, as well as to bleomycin and antiseptics and disinfectants (Berg et al., 1998; Liu et al., 2013). The pSK41 Rep protein (319 aa) has been divided into three functional domains. The N-terminal 120 residues (NTD) contains the conserved RepA_N domain and mediates *ori* specific binding at four Rep boxes found centrally within the *rep* gene (Kwong et al., 2004; Liu et al., 2012). The NTD also contains sequences necessary for oligomerization of the protein (Schumacher et al., 2014). The central domain (121–199 aa), encoded by DNA sequences corresponding to the *ori*, functions as a linker and proteins carrying an in-frame deletion of the central domain can rescue replication of Rep-defective plasmids containing a functional *ori* (Liu et al., 2012). The Rep C-terminal domain (CTD; 200–319) is essential for replication and displays considerably high sequence conservation, but only in plasmids from the same genera, suggesting that it may perform a host-specific function (Weaver et al., 2009). Recently, it was shown that the pSK41 Rep CTD interacts directly with the *S. aureus* DnaG primase (Schumacher et al., 2014).

Crystallographic analysis of RepA_N proteins indicated that the Rep NTD readily forms tetramers and contains a winged HTH that allows interaction with both the major and the minor grooves of Rep box DNA, inducing a bend of approximately 30° (Schumacher et al., 2014). This was consistent with the intrinsic bend associated with A+T-rich tracts and substitution of this Rep box sequence to a G+C-rich tract reduced pSK41 Rep binding by 10-fold (Schumacher et al., 2014). The Rep CTD was shown to form a compact structure composed of five helices and on its own was monomeric. Interestingly, the pSK41 Rep structures of the NTD and CTD were found to display structural similarity to the primosomal protein DnaD, suggesting a common evolutionary origin (Schumacher et al., 2014). Structural similarity between the *B. subtilis* DnaD and DnaB proteins that was not evident in pairwise sequence alignments has also been described (Marston et al., 2010). In *B. subtilis*, DnaD and DnaB play a central role in both DnaA-mediated initiation of replication at *oriC* and also in restart of stalled replication forks by primosome assembly (Bruand et al., 1995; Smits et al., 2011).

Multiple incompatibility groups are likely to exist within the staphylococcal RepA_N plasmids since pSK41 and pSK1, which encode divergent RepA_N proteins, are known to be compatible (Firth et al., 2000). A novel chimeric replication initiation gene was identified in the high-level mupirocin resistance plasmid pPR9 from Spain (Pérez-Roth et al., 2010). pPR9 displays high-level nucleotide sequence homology to pSK41 throughout the backbone including replication, maintenance, and transfer regions, except for a ∼500-bp region corresponding to the pSK41 RepA_N domain and replication origin (Pérez-Roth et al., 2010). The pPR9 Rep NTD was instead found to share homology to a putative phage replication protein while retaining > 97% amino acid sequence identity to the pSK41 Rep CTD and 99% nucleotide sequence identity in the upstream *rep* control region. Instead of the winged-HTH present in the RepA_N domain, the pPR9 Rep NTD contains a putative HTH belonging to pfam13730. Construction of a pPR9 mini-replicon showed that the pPR9 *rep* region supported autonomous replication and that it was compatible with a pSK41 mini-replicon (Pérez-Roth et al., 2010). This intriguing modular pPR9 initiator represents a new initiator type that could have evolved to overcome incompatibility barriers. This type of hybrid initiation gene is found in a small number of other plasmids such as pUSA03 from caMRSA strain USA300 (Diep et al., 2006).

### Rep_3 Plasmids

One fifth of theta-replicating *S. aureus* plasmids were found to carry a replication initiation gene that gives rise to a product containing the conserved Rep_3 domain (pfam01051). In staphylococci, this type of initiator was first observed on the small (8 kb) *S. epidermidis* trimethoprim resistance plasmid pSK639 (Apisiridej et al., 1997). The pSK639 *rep* gene encodes a protein of 287 residues in length with the conserved Rep_3 domain spanning the first ∼220 residues and a ∼50 residue CTD. Like the RepA_N plasmids, Rep_3 domain initiators are widely distributed in plasmids from low G+C Gram-positive bacteria including coagulase-positive and -negative staphylococci, enterococci, and lactococci and are distantly related to a large number of iteron-regulated plasmid initiators of Gram-negative bacteria including *Pseudomonas syringae* plasmid pPS10, *Escherichia coli* plasmids F, pSC101, R6K, P1, and broad host range plasmid RK2. The Gram-negative Rep_3 initiators are variously dependent upon DnaA for their replication and often contain dimerization motifs critical for copy number control (Ingmer et al., 1995; Matsunaga et al., 1997; del Solar et al., 1998; Toukdarian and Helinski, 1998; Das and Chattoraj, 2004; Giraldo and Fernández-Tresguerres, 2004; Swan et al., 2006; Konieczny et al., 2014).

Upstream of the pSK639 *rep* coding region, in the vicinity of the promoter, is a series of five, 22 bp tandemly repeated sequences that most likely represent Rep binding sites and constitute the origin of replication (Apisiridej et al., 1997). The position of these potential Rep binding sites suggests that pSK639 Rep may autoregulate its own transcription (Apisiridej et al., 1997) although this has yet to be demonstrated. Iteron-mediated regulation has been shown to be the primary copy number control mechanism in many of the Rep_3 domain plasmid replicons from Gram-negative bacteria and could also play a role in pSK639 copy number regulation. This form of regulation relies upon dimerization domains that allow plasmids to pair (handcuff). In these plasmids, Rep proteins are only active for initiation as monomers and at higher Rep concentrations dimerization promotes plasmid pairing and the inhibition of replication initiation.

### PriCT_1 Plasmids

A smaller number of staphylococcal theta-replicating plasmids (∼6%) encode a replication initiator belonging to the broad host range Inc18 family, which includes the enterococcal conjugative plasmid pAMβ1 and streptococcal conjugative resistance plasmids pSM19035 and pIP501. The three plasmids share a high degree of sequence identity (Lioy et al., 2010) and utilize closely related Rep proteins containing the conserved PriCT_1 domain (**Figure 2**), which are considerably larger than most plasmid replication initiators (∼60 kDa). Staphylococcal plasmids that were detected to contain a PriCT_1 type replication initiator include pCH91 (17 kb), which encodes a type II toxin-antitoxin system *pemIK* (Bukowski et al., 2013), the exfoliative toxin B plasmid pETB (38 kb; Yamaguchi et al., 2001), pWBG707 (Udo et al., 1992), and *cfr*-carrying conjugative plasmid pSA737 (39 kb; Mendes et al., 2013). The other PriCT_1 plasmids detected in *S. aureus* were also found to encode a RepA_N or Rep_3 initiator gene.

Replication of Inc18 plasmids has been characterized in considerable detail using pAMβ1 to study the replication ini-tiation mechanism and pIP501 the copy number control mecha-nism. These plasmids exhibit a broad host range. In pAMβ1 replication, the RepE monomer binds to a single 25-bp sequence in the origin, which is located immediately downstream of the *repE* gene and induces localized melting of a short DNA region (15 nt) found next to the binding site (Le Chatelier et al., 2001). The RepE protein has higher affinity for non-specific single-stranded DNA than for its double-stranded binding site and this activity is believed to play a role in extending *ori* strand opening. Transcription through the *ori* is essential for the replication process, which is independent of DnaA but requires DNA polymerase I (Bruand et al., 1993; Ceglowski et al., 1993; Bruand and Ehrlich, 1998). It has been proposed that the Rep transcript synthesized by RNAP stalls at *ori* when Rep is bound, the transcript is cleaved (by RNAP or Rep) leaving a ∼20-nt RNA that acts as the replication primer, which is extended by DNA polymerase I (Le Chatelier et al., 2001). The D-loop structure generated by DNA polymerase I is then an efficient substrate for PriA-mediated primosome assembly that requires the host-encoded replication proteins DnaB, DnaD, and DnaI (Polard et al., 2002). Sequences central to the pAMβ1 *ori* (5′-TGCCATTACATTTAT-3′) that constitute the RepE binding site (Le Chatelier et al., 2001) and are also found in the minimal *ori* of pIP501 and pSM19035 (Brantl and Behnke, 1992a; Lioy et al., 2010) can be detected in an analogous position downstream of the *rep* genes in staphylococcal plasmids pCH91, pETB, pWBG707, and pSA737, suggesting that they utilize a similar replication initiation mechanism. Furthermore, *cop* and antisense RNA genes similarly positioned to copy number control elements in pIP501 (see below; Brantl and Behnke, 1992b) can also be detected in each of the staphylococcal plasmids.

## Copy Number Control Mechanisms

Antisense RNA-mediated copy number control is broadly utilized by both RCR and theta-replicating staphylococcal plasmids where regulation of replication has been investigated, including members of the pT181, pE194, pC194, and pSK41 families. Copy number control in the pSN2, pSK639, and pCH91 families have not yet been studied in detail, however, as described above, members of the latter family appear likely to utilize copy number control systems similar to the Inc18 broad host range conjugative pIP501 employing both a small protein repressor (Cop) and an antisense RNA-mediated attenuation system to regulate Rep expression (reviewed in Brantl, 2014 and Grohmann et al., 2016). Dual-regulation of copy number by both Cop repressor and antisense RNA has also been well established in members of the pE194 family via pMV158 (del Solar and Espinosa, 2000), although the mechanistic details of the systems are distinctive for each family.

### Antisense RNA-Mediated Copy Number Control of Rep_trans Plasmids

pT181 family plasmids use small, untranslated antisense RNAs to regulate expression of the Rep protein and thereby control replication initiation. In pT181, the 87-nt antisense RNA (RNAI) is counter-transcribed to *repC* and is complementary to the *repC* mRNA untranslated leader region. RNA–RNA interaction between RNAI and the *repC* mRNA causes the formation of a thermodynamically stable secondary structure (stem-loop IV) immediately upstream of the *repC* start codon (Novick et al., 1989). Stem-loop IV is predicted to contain the ribosome binding site, however, the main effect of antisense RNA binding is transcriptional termination at stem-loop IV (which resembles a σ-independent terminator) and the attenuated transcripts are incapable of producing RepC (Novick et al., 1989). In the absence of RNAI, sequences in the 5′-proximal arm of stem-loop IV preferentially pair with another complementary sequence in the *repC* leader, termed the pre-emptor, preventing formation of stem-loop IV and allowing full-length *repC* mRNA to be transcribed (Novick et al., 1989).

### Copy Number Control in Rep_2 Family Plasmids

pE194 family plasmids are predicted to utilize a copy number control system that has been studied extensively in pMV158 and its deletion derivative pLS1. pMV158 encodes two *trans*-acting negative regulators of the replication initiation gene, *repB*, an antisense RNA (RNAII) and a small repressor protein (CopG; del Solar and Espinosa, 1992, 2000). *copG* is found upstream of *repB* and the two genes form an operon. RNAII is a 48-nt transcript that is counter-transcribed from a promoter within the 5′-end of the *repB* coding sequence and is complementary to a region found immediately upstream of an atypical ribosome binding site essential for RepB translation (López-Aguilar et al., 2013). It has been proposed that mRNA-RNAII duplex formation hinders binding of the ribosome to the translation initiation region (López-Aguilar et al., 2013). RNA–RNA interactions initiate through base contacts in the RNAII 5′ single-stranded tail, while the RNAII stem-loop appears to only play an auxiliary role in RepB translational repression (López-Aguilar et al., 2015). CopG is dimeric in solution and has a ribbon-helix-helix structure (RHH_1, pfam01402; Gomis-Rüth et al., 1998). It has been shown that four dimers bind cooperatively to the *copG* promoter leading to transcriptional repression of the *copG*-*repB* transcript by competitively inhibiting the RNAP-promoter interaction (Hernández-Arriaga et al., 2009). A recent study has indicated ‘crosstalk’ between the pMV158 mobilization and replication systems. MobM was found to bind and repress the RNAII promoter, leading to elevated levels of RepB and an increase in plasmid copy number (Lorenzo-Díaz et al., 2017). Staphylococcal pE194 (Rep_2) family plasmids including pCPS49, pDLK3, SAP085B each have analogously positioned elements that are predicted to give rise to a CopG-like repressor and a RNAII-like antisense RNA. Thus, the staphylococcal plasmids carrying a Rep_2 initiator are all expected to utilize similar copy number control system to pMV158. pE194, pCPS49 and SAP085B also carry a *mob/pre* gene distantly related to pMV158 *mobM*. In Rep_1 family plasmids pC194 and pUB110, antisense RNAs are also predicted to be counter-transcribed in the respective Rep translation initiation regions and have been proposed to directly block translation of the initiator (Alonso and Tailor, 1987; Maciag et al., 1988), although the intricacies of these antisense RNA-mediated control systems have yet to be described in the same detail.

### Antisense RNA-Mediated Copy Number Control of RepA_N Plasmids

Staphylococcal RepA_N plasmids that have so far been examined are found to employ closely related antisense RNA-mediated copy number control systems, comparable to the prototype pSK41 (Kwong et al., 2008). It is noteworthy that similar RNA-mediated control systems do not appear to be present in RepA_N plasmids from other genera. Expression of pSK41 Rep is negatively regulated by a ∼83-nt antisense RNA (RNAI) that is counter-transcribed to the *rep* mRNA and is complementary to its leader region in a position ∼100 nt upstream of the translation initiation region (Kwong et al., 2004). It has been proposed that binding of RNAI to the Rep mRNA leader induces formation of a stem-loop secondary structure in the *rep* translation initiation region. However, unlike pT181 family plasmids, the antisense RNA induced stem-loop does not attenuate transcription but rather sequesters the ribosome binding site in the stem-loop rendering it inaccessible to the translation machinery (Kwong et al., 2006). Secondary structure probing of pSK41 RNAI revealed the presence of two stem-loops separated by an 8-nt single-stranded spacer and an unstructured 18-nt 5′-tail (Kwong and Firth, 2015). Mutations in either stem-loop significantly reduced RNAI repressor activity but the single-stranded regions could be deleted without affecting RNAI function (Kwong and Firth, 2015), indicating that complete base pairing between the antisense RNA and its target was not required.

### Copy Number Control in PriCT_1 Plasmids

pIP501 copy number is controlled by two *trans*-acting negative regulators, a small repressor protein, CopR, and an antisense RNA, designated RNAIII (see **Figure 1**; pCH91). *copR* is found upstream of the initiator gene, *repR*, but the genes are independently transcribed. CopR consists of 92 amino acid residues and contains a conserved HTH domain (HTH_XRE; pfam01381) that facilitates operator DNA binding as a dimer (Steinmetzer et al., 1998). CopR does not autoregulate but binds and represses transcription from the *repR* promoter (Brantl, 1994). It has also been demonstrated that CopR-mediated repression of the *rep* promoter effectively increases expression of RNAIII by preventing convergent transcription (Brantl and Wagner, 1997). RNAIII interacts with the leader of the RepR mRNA to induce the formation of a terminator-like structure that results in attenuation of the RepR transcript in a similar manner to that observed in pT181 replication control (Brantl et al., 1993). In *S. aureus* plasmids pCH91, pWBG707, and pSA737 all of the copy number control elements present in pIP501 can be detected even though the predicted replication initiators of the staphylococcal plasmids only share ∼30% amino acid sequence identity to pIP501 RepR. These include a small HTH domain protein of the XRE family (Cop), a *rep* promoter that gives rise to a long (∼320 nt) leader, an antisense RNA promoter positioned midway through the leader that could give rise to an antisense RNA (**Figure 2**), and inverted repeats followed by a poly[T] tract just 5′ of the *rep* translation initiation region that appears capable of forming a σ-independent terminator-like structure. The presence of these elements indicates that the staphylococcal PriCT_1 family plasmids are likely to use an analogous copy number control system to plasmid pIP501.

## Host-Encoded Proteins In Plasmid Replication

As we have described above, plasmids encode their own replication components for the initiation of replication, including a replication initiation protein and an origin of replication, and a mechanism that controls the expression/activity of the initiation protein. The interaction between initiator and origin prepares the DNA for replication, either by strand-specific cleavage at *dso* (generating a 3′-OH) or melting of strands at *ori*. Both of these replication mechanisms then depend on helicase enzymes to facilitate further duplex melting. In contrast to RCR plasmids, and as part of the initiation process, theta-replicating plasmids require synthesis of a leading strand replication primer to generate 3′-OH. Once initiated, plasmids then rely on replisomes consisting of host-encoded replication proteins that are normally used for chromosomal replication and repair. In theta-replication of plasmids and the chromosome, the replisome is composed of DNA polymerase III holoenzyme, primase, sliding clamps, helicase, and other accessory factors (Kornberg and Baker, 1992). Fundamental differences between the RC replication mechanism and theta-type replication mechanism (assymetric vs. semi-conservative) suggest that the replisome components could be quite different. In this section, we discuss some of the host-encoded proteins that are known to play a role in the replication of staphylococcal plasmids.

In most bacteria, DnaA is the essential replication initiator of the chromosomal origin, *oriC*. In many theta-replicating plasmids of *E. coli*, the Rep proteins have been shown to recruit DnaA to their *ori* to assist in the initiation step and often the *ori*s possess DnaA boxes homologous to DnaA-binding sites in *oriC* (del Solar et al., 1998). DnaA has not yet been directly implicated in the replication mechanism of any staphylococcal plasmids and plasmid DnaA boxes have not so far been detected.

### Polymerases

*Escherichia coli* possesses five different DNA polymerases, Pols I, II, III, IV, and V. Pols II, IV, and V are translesion polymerases, Pol III is the core processive polymerase involved in the replisome and PolI is required in lagging strand theta-replication to remove RNA primers and fill in the gaps of Okazaki fragments (Kornberg and Baker, 1992). Low G+C, Gram-positive bacteria usually possess three DNA polymerase enzymes, PolC, DnaE, and PolA, which are thought to be functionally equivalent to PolIII, PolII, and PolI, respectively. DnaE has been shown to be essential for viability in both *S. aureus* and *B. subtilis* (Dervyn et al., 2001; Inoue et al., 2001). In *B. subtilis*, DnaE was not involved in leading strand synthesis but was essential in lagging strand synthesis for initial extension of RNA primers (Sanders et al., 2010). The role of PolA would likely be in removing RNA primers and joining Okazaki fragments as in *E. coli*. PolA has also been shown to be involved in specific stages of replication of some staphylococcal plasmids including lagging strand synthesis of RCR plasmids (Diaz et al., 1994; Kramer et al., 1997) and initial extension of the leading strand RNA primer in PriCT_1-family plasmids (Bruand et al., 1993). In both of these stages of plasmid replication, RNAP is involved in generating the replication primer at the *sso* or by transcription through *ori* as described above.

### Helicases

Bacteria have multiple helicases that have specialized roles (Hall and Matson, 1999). In *E. coli*, DnaB helicase is the primary replicative helicase and is required for replication of the chromosome and theta-replicating plasmids (Kornberg and Baker, 1992). Specialized helicases include the misleadingly named “Rep” helicase protein involved in the replication of some phages (Lane and Denhardt, 1975; Takahashi et al., 1979), and UvrD that engages in DNA repair and replication of some viruses and RCR plasmids (Bierne et al., 1997; Bruand and Ehrlich, 2000). In low G+C Gram-positive bacteria, the DnaB homolog, DnaC, is expected to fulfill the main replicative helicase role.

Rolling-circle replication plasmids of the pT181 family have been shown to require the host-encoded helicase PcrA for replication. Mutations in *S. aureus pcrA* led to the accumulation of pT181 initiation complexes indicating that a transition to elongation phase of replication had stalled (Iordanescu and Basheer, 1991). Suppressor mutations that restored replication were mapped to the pT181 Rep protein, suggesting a direct interaction between the two proteins (Iordanescu, 1993). Rep loads the helicase onto the lagging strand of the nicked *dso* and remains engaged with PcrA increasing its processivity and enabling it to displace DNA from a nicked substrate (Soultanas et al., 1999; Chang et al., 2002; Anand and Khan, 2004; Zhang et al., 2007). pT181 Rep displays an interaction with the PcrA helicases of *S. aureus, Bacillus anthracis, Bacillus cereus*, and *Streptococcus pneumoniae* but fails to stimulate full unwinding activity in the latter (Anand et al., 2004; Ruiz-Masó et al., 2006). Together with previous observations that pT181 can replicate in bacilli (albeit unstably) but not streptococci, these results indicate that Rep-mediated activation of PcrA is a requirement for efficient replication and lack of interaction is likely to limit plasmid host range. In *B. subtilis* carrying the *pcrA3* mutation, pT181 was incapable of replication; however, pC194 and pE194 plasmids could still replicate at normal copy number. This suggested that either another helicase may be required for leading strand synthesis in pC194 and pE194 or that the *pcrA3* mutation does not effect PcrA interaction with their respective Rep proteins (Petit et al., 1998). Interestingly, in *E. coli* host cells, the PcrA homolog UvrD was found to be essential for pC194 and pE194 replication (Bruand and Ehrlich, 2000).

### Primases

The bacterial primosome is a multiprotein complex containing helicase, primase, and accessory proteins that assist in helicase loading and is required for generating RNA primers on single-stranded DNA. Primosomes are assembled during the initiation of chromosome replication at *oriC* (DnaA-dependent) and in the restart of stalled or collapsed replication forks (PriA-dependent). In Gram-positive bacteria, the replicative helicase (DnaC) is loaded through DnaI with the assistance of DnaB and DnaD. Once DnaC has been loaded it recruits DnaG primase and this primosome complex may then associate with the PolC holoenzyme and other factors to constitute the replisome. pAMβ1 carries a primosome assembly site (*ssiA*) downstream of *ori* that requires PriA, DnaB, DnaD, and DnaI suggesting that the activated *ori* (containing a D-loop) is recognized and targeted by PriA in a process that resembles re-combinational DNA repair (Polard et al., 2002). pSK41 Rep was shown to share structural similarity to DnaD primosomal helicase loader and interact directly with DnaG primase (Schumacher et al., 2014). Thus, it is possible that in pSK41, Rep assists loading of the DnaC helicase, perhaps in combination with DnaB and DnaI, or it may recruit DnaC indirectly through helicase interaction domains conserved in DnaG.

## Concluding Remarks

The different types of plasmid replication systems described here encompass the diversity of plasmids recognized in staphylococci. Plasmids using each of these systems have been shown to act as vehicles for the carriage of antimicrobial resistance genes. Our view of plasmid diversity in staphylococci is heavily skewed by a historical focus on clinical isolates, and the consequential bias toward *S. aureus* and hence under-representation of coagulase negative species. The extent to which current understanding represents a comprehensive or distorted description of the staphylococcal plasmidome is an open question that awaits far broader sampling of the disparate environments occupied by staphylococci. Advances in sequencing capacity provide an opportunity to address this knowledge gap, while increasing evidence of transmission pathways linking bacteria that impact human health with those in the broader biosphere should provide motivation.

Just as our understanding of plasmid diversity is likely incomplete, the level to which the differing replication systems used by staphylococcal plasmids have been studied varies tremendously. While RCR plasmid replication has been analyzed in considerable detail, the replication systems of theta-replicating plasmids have been largely ignored in comparison, despite the established significance of these plasmids in the expression of resistance and virulence properties. There are several areas where information about these larger staphylococcal plasmids is particularly lacking. This includes how their replication systems interface with the chromosomally encoded replication machinery, and how they integrate and cooperate with other plasmid modules associated with plasmid propagation, such as partitioning, conjugation, and mobilization systems. There seems to be a general view that plasmid biology is well understood, but this is really not the case excepting a handful of model systems. This point has been highlighted by the recent characterization of new types of staphylococcal conjugative plasmids and identification of previously unrecognized mobilization determinants that are widespread on staphylococcal plasmids (O’Brien et al., 2015; Ramsay et al., 2016; Ramsay and Firth, 2017). Given the pivotal role plasmids play in bacterial adaptation, not least in the emergence of staphylococcal resistance, a renewed emphasis on studies elucidating the properties and mechanisms of plasmids is required if we are to meaningfully appreciate their roles in bacterial evolution and its consequences.

## Author Contributions

All authors listed have made a substantial, direct and intellectual contribution to the work, and approved it for publication.

## Conflict of Interest Statement

The authors declare that the research was conducted in the absence of any commercial or financial relationships that could be construed as a potential conflict of interest.
