# Supplementary material for: Replication of Staphylococcal Resistance Plasmids
Source: Front Microbiol. 2017 Nov 23;8:2279. doi: 10.3389/fmicb.2017.02279 (PMC5703833; doi:10.3389/fmicb.2017.02279)
Supplement: Supplementary file 1 [file Data_Sheet_1.PDF]

Conjugativ e	Plasmid name	Accession	GI numbers	SIZE	Rep-AN like RepA_N	pMW2 rep_3 Rep_3	RepE (pETB) PriCT_1	RepF Rep_2 li Rep_2	RepC Rep_trans	Rep_1 superfa Rep_1 superfa	RepL RepL	Conjugativ e
	277											
No	pSN2	NC_005565.1	42761428	1,288								No
No	SAP093B	NC_013310.1	410108954	1,290								No
No	pDLK3	NC_013969.1	292657203	1,365								No
No	pAVY	NC_013451.1	262225764	1,442								No
No	pSK6	NC_001995.1	10956171	1,551								No
No	SAP104B	GQ900451.2	282166108	1,552								No
No	pSK3	NC_001994.1	10956168	1,658								No
No	pNVH99	AJ296103.1	11124600	2,239								No
No	pWBG754	GQ900396.1	260066098	2,241								No
No	pLUH01	NC_017346.1	384863394	2,241								No
No	TCH70_1	NZ_ACHH02000015.1	300912861	2,300								No
No	pS1e	NZ_AUPS01000034.1	544872643	2,362								No
No	A6300	NZ_ACKF01000018.1	258445547	2,366								No
No	p9b	NC_019143.1	410655971	2,367								No
No	SAP087A	GQ900439.1	260066141	2,396								No
No	pWBG764	GQ900468.1	260066170	2,397								No
No	A9765	NZ_ACSN01000068.1	282929330	2,399								No
No	A9719	NZ_ACKJ01000014.1	258420073	2,402								No
No	pDLK1	NC_019139.1	410655957	2,402								No
No	pKH20	NC_010686.1	188039005	2,412								No
No	SAP078B	GQ900431.1	260066133	2,415								No
No	pKH8	U50077.1	1236637	2,417								No
No	Cn1	NC_022228.1	537462731	2,472								No
No	pE5	M17990.1	153063	2,473								No
No	pWBG738	NC_007209.1	71558996	2,473								No
No	pKH19	NC_010685.1	188039008	2,473								No
No	CF-Marseill	NZ_CABA01000093.1	221079778	2,473								No
No	pWBG751	GQ900393.1	260066095	2,473								No
No	p19321-P0	NC_018969.1	410655417	2,473								No
No	pT48	NC_001395.1	9507387	2,475								No
No	TCH130	NZ_ACHD01000266.1	224922483	2,476								No
No	TCH959	NZ_AASB02000192.1	225353765	2,476								No
No	pKH4	U81980.1	1848267	2,487								No
No	pKH21	NC_010684.1	188039011	2,531								No
No	A5948_3	NZ_ACKD01000027.1	258451705	2,562								No
No	pS1d	NZ_AUPS01000033.1	544872642	2,649								No
No	pNVH01	NC_004562.1	28261693	2,650								No
No	pUR5425	NC_019146.1	410655987	2,690								No
No	pBMSa1	AY541446.1	44985410	2,750								No
No	pSA1308	NC_007928.1	90969145	2,756								No
No	pKH15	NC_010427.1	170780406	2,907								No
No	SAP065A	GQ900420.1	260066122	2,908								No
No	pDLK2	GU562625.1	291032728	2,908								No
No	pBMb9393	NC_021657.1	521147365	2,908								No
No	pKH13	NC_010426.1	170780403	2,909								No
No	pC194	NC_002013.1	10956139	2,910								No
No	pWBG1773	NC_010616.1	187729625	2,916								No
No	pKH3	NC_005020.1	32470383	2,979								No
No	pLGA251	NC_017348.1	387781658	2,993								No
No	pKH12	NC_010687.1	188039002	3,011								No
No	SAP070A	GQ900423.1	260066125	3,011								No
No	pTW20_2	FN433598.1	269942329	3,011								No
No	SAP104A	GQ900450.2	282166104	3,011								No
No	pKH14	NC_010428.1	170780409	3,124								No
No	pUSA01	NC_007790.1	87159837	3,125								No
No	SAP046B	GQ900404.1	260066106	3,125								No
No	SAP049B	GQ900408.1	260066110	3,125								No
No	SAP051B	GQ900411.1	260066113	3,125								No
No	p18807-P0	CP002136.1	304381986	3,125								No
No	p18808-P0	CP002138.1	304382750	3,125								No
No	p18811-P0	CP002144.1	304386687	3,125								No
No	pS0385-3	AM990995.1	283471940	3,158								No
No	pKH18	NC_010231.1	164564304	3,332								No
No	pC55s	AY048756.1	15777858	3,333								No
No	pNewBould	NZ_AKYW01000028.1	421151015	3,379								No
No	pE194	NC_005908.1	49489772	3,728								No
No	pSBK203	U35036.1	1015405	3,780								No
No	pNS1	M16217.1	150832	3,879								No
No	pS1c	NZ_AUPS01000031.1	544872637	3,899								No
No	SAP085B	GQ900438.1	260066140	3,961								No
No	pKH7	NC_002096.1	10956155	4,118								No
No	C101_1.1	NZ_GG730120.1	282922259	4,278								No
No	M899	NZ_GG730190.1	282912712	4,368								No
No	pS0385-2	AM990994.1	283471934	4,381								No
No	pS194	NC_005564.1	42761423	4,397								No
No	pS123b	NZ_AUPU01000024.1	544872931	4,397								No
No	pWBG760	GQ900473.1	260177099	4,412								No
No	pKH6	U38428.1	1052997	4,439								No
No	pUSA02	NC_007791.1	87159843	4,439								No
No	SAP085A	GQ900437.1	260066139	4,439								No
No	SAP093A	GQ900441.1	260066143	4,439								No
No	SAP094A	GQ900443.1	260066145	4,439								No
No	SAP095A	GQ900445.1	260066147	4,439								No
No	pT181	NC_006629.2	77102894	4,440								No
No	pKH17	NC_010284.1	166153459	4,441								No
No	pKH16	NC_010262.1	165975441	4,442								No
No	ATCC BAA-	NZ_AEEK01000040.1	304380005	4,457								No
No	A8115_con	NZ_ACKG01000002.1	258440600	4,498								No
No	SAP060B	GQ900417.1	260066119	4,498								No
No	MR1_139	NZ_ACZQ01000139.1	295823321	4,522								No
No	pUB110	NC_001384.1	9507338	4,548								No
No	JKD6009	NZ_ABSA01000025.1	194045651	4,548								No
No	A6300_2	NZ_ACKF01000048.1	258447669	4,554								No
No	A10102	NZ_ACSO01000036.1	282929825	4,554								No
No	A9763	NZ_ACKK01000035.1	258416007	4,555								No
No	A6244	NZ_ACKE01000028.1	258448810	4,555								No
No	pTZ4	NC_010111.1	162145842	4,555								No
No	pC221	NC_006977.1	211774469	4,555								No
No	CF-Marseill	NZ_CABA01000045.1	221079730	4,556								No
No	A5937	NZ_ACKC01000002.1	258453350	4,556								No
No	SAP084A	GQ900436.1	260066138	4,595								No
No	SAP089A	NC_013308.1	410108952	4,595								No
No	MR1_138	NZ_ACZQ01000138.1	295823320	4,604								No

No	pC223	AY355285.1	33868600	4,608		No
No	pKKS49	NC_019149.1	410656020	4,809		No
No	pS1b	NZ_AUPS01000028.1	544872628	4,810		No
No	A9754	NZ_ADJI01000035.1	294850799	4,947		No
No	58-424_1	NZ_GG749054.1	293495419	5,143		No
No	pS0385-1	AM990993.1	283471929	5,246		No
No	pCPS49	NC_019142.1	410655967	5,292		No
No	pS94a	NZ_AUPW01000021.1	544873194	5,442		No
No	pVGA	NC_011605.1	215401543	5,713		No
No	pCPS32	NC_019141.1	410655963	5,718		No
No	pJ3358	NC_001763.1	10956144	6,024		No
No	pUR3912	NC_020183.2	507527156	6,176		No
No	pKKS627	NC_014156.1	296274672	6,242		No
No	pSA8589	NC_021230.1	502045013	6,962		No
No	pMSA16	NC_019144.1	410655974	7,054		No
No	pUR4128	NC_019147.1	410655990	7,567		No
No	pUR2355	NC_019145.1	410655980	7,609		No
No	pRJ6	NC_011522.1	212383450	7,904		No
No	pS130a	NZ_AUPT01000023.1	544872828	8,882		No
No	pSK17	GQ900513.1	260066065	9,968		No
No	pRJ9	AF447813.1	25045961	10,406		No
No	SAP077B	NC_013341.1	260667563	13,342		No
No	pKKS832	FN377602.2	295980134	14,362		No
No	ATCC 5181:	NZ_ADVP01000034.1	297208859	16,164		No
No	SAP099B	GQ900449.1	260066151	16,428		No
No	SAP105B	NC_013378.1	260874699	16,775		No
No	pAVX	NC_013453.1	262260515	17,256		No
No	pBORa53	NC_013550.1	270208715	17,334		No
No	pCH91	NC_020227.1	448819501	17,515		No
No	A8796	NZ_ADJJ01000025.1	297246679	17,758		No
No	TCH70_2	NZ_ACHH02000018.1	300913061	18,140		No
No	pSA268	NC_023278.1	571043561	20,269		No
No	pUSA300H(	NC_010066.1	161508245	20,413		No
No	55/2053	NC_022126.1	532438800	20,449		No
No	SAP055A	GQ900414.1	260066116	20,453		No
No	pSAS	BX571858.1	49168429	20,652		No
No	pWBG750	GQ900392.1	260066094	20,653		No
No	SAP072A	GQ900424.1	260066126	20,653		No
No	pMW2	NC_005011.1	32470355	20,654		No
No	SAP053A	GQ900413.1	260066115	20,672		No
No	p21	NC_002517.1	10956174	20,719		No
No	SAP073A	GQ900425.1	260066127	20,729		No
No	pWBG757	GQ900397.1	260066099	20,730		No
No	pWBG763	GQ900467.1	260066169	20,730		No
No	pSaa6159	CP002115.1	302334329	20,730		No
No	pUB101	NC_005127.1	37595761	21,845		No
No	65-1322	NZ_GG700565.1	257428637	22,093		No
No	p11819-97	NC_017350.1	385782932	22,317		No
No	SAP058A	GQ900479.1	260177105	22,439		No
No	pSK62	GQ900446.1	260066148	22,694		No
No	pSA1379	NC_007931.1	90969148	22,787		No
No	SAP060A	GQ900416.1	260066118	22,987		No
No	A8115_con	NZ_ACKG01000021.1	258443280	22,988		No
No	SAP051A	GQ900410.1	260066112	23,059		No
No	M876	NZ_GG700623.1	257436870	23,214		No
No	E1410	NZ_GG700609.1	257433966	23,259		No
No	A8117	NZ_ACYO01000019.1	282895185	23,292		No
No	M1015	NZ_GG749015.1	293550561	23,501		No
No	M809	NZ_GG749324.1	293511852	23,537		No
No	pSK76	GQ900444.1	260066146	23,983		No
No	C101_1.21	NZ_GG730140.1	282926050	24,019		No
No	WBG10049	NZ_GG730219.1	282912686	24,446		No
No	pWBG756	GQ900472.1	260177098	24,456		No
No	TCH60_unn	NC_017345.1	384868560	24,491		No
No	58-424_3	NZ_GG749073.1	293509085	24,634		No
No	pN315	NC_003140.1	16119200	24,653		No
No	A9635	NZ_ACKI01000002.1	258422557	24,653		No
No	CA-347	NC_021552.1	514064933	24,653		No
No	pWBG752	GQ900394.1	260066096	24,654		No
No	pST75	NC_016942.1	379793806	24,853		No
No	SAP049A	GQ900407.1	260066109	25,022		No
No	VRSAp	NC_002774.1	14141823	25,107		No
No	pWBG755	GQ900471.1	260177097	25,607		No
No	pPM1	NC_019148.1	410655997	25,961		No
No	SAP063A	GQ900418.1	260066120	26,016		No
No	SAP105A	NC_013377.1	260874698	26,236		No
No	SAP059A	GQ900480.1	260177106	26,243		No
No	SAP065B	GQ900481.1	260177107	26,750		No
No	pWBG761	GQ900474.1	260177100	26,838		No
No	SAP071A	GQ900485.1	260177111	26,883		No
No	p18807-P0:	CP002135.1	304381946	26,972		No
No	pUSA300H(	NC_010063.1	161510924	27,041		No
No	p18808-P0:	CP002137.1	304382043	27,059		No
No	SAP050A	GQ900409.1	260066111	27,067		No
No	p18810-P0:	NC_018963.1	410655288	27,067		No
No	SAP015A	GQ900380.1	260066082	27,068		No
No	SAP046A	GQ900403.1	260066105	27,068		No
No	p18806-P0:	CP002134.1	304381909	27,068		No
No	pLAC-P03	CP002149.1	304389212	27,068		No
No	p18805-03	CP002132.1	304380339	27,069		No
No	p18811-03	CP002143.1	304386437	27,070		No
No	68-397	NZ_GG700590.1	257431573	27,097		No
No	SAP056A	GQ900478.1	260177104	27,128		No
pWBG749 t	pM0408	AIWO01000029.1	477787193		27,223	pWBG749 type
No	SAP012A	GQ900377.1	260066079	27,267		No
No	A9781	NZ_ACKL01000034.1	256402493	27,268		No
No	pWBG744	GQ900398.1	260066100	27,268		No
No	SAP048A	GQ900406.1	260066108	27,268		No
No	SAP074A	GQ900426.1	260066128	27,268		No
No	pLUH02	FR714929.1	312831055	27,271		No
No	pZ172_1	NC_022610.1	554652311	27,326		No
No	p19231-P0:	CP002147.1	304388562	27,425		No
No	SAP019A	GQ900385.1	260066087	27,435		No
No	pSK67	NC_019010.1	410108987	27,439		No
No	pSK77	GQ900494.1	260177120	27,694		No
No	A8819	NZ_ADJK01000020.1	295407577	27,798		No
No	pSK1	NC_014369.1	302344722	28,150		No
No	pWBG759	GQ900401.1	260066103	28,384		No
No	p18809-P0	CP002146.1	304388018	28,404		No

No	SAP047A	NC_013331.1	260667553	28,974		No
No	pI258	GQ900378.1	260066080	29,254		No
No	SAP103A	GQ900497.1	260177123	29,258		No
No	SAP104C	GQ900498.1	260177124	29,343		No
No	SAP070B	GQ900484.1	260177110	29,531		No
No	pSK59	GQ900488.1	260177114	29,570		No
No	pTW20_1	FN433597.1	269942301	29,585		No
No	SAP027A	GQ900388.1	260066090	29,646		No
No	pWBG753	GQ900395.1	260066097	30,047		No
pSK41 type	p18813-P0:	NC_018967.1	410655332		30,127	pSK41 type
No	pSJH901	NC_009477.1	148244139	30,429		No
No	pSJH101	NC_009619.1	150375692	30,429		No
No	SAP064A	GQ900419.1	260066121	30,857		No
No	pSK57	GQ900493.1	260177119	31,164		No
No	pSK80	GQ900492.1	260177118	31,314		No
No	SAP066A	GQ900482.1	260177108	32,122		No
No	SAP101A	GQ900495.1	260177121	32,163		No
No	pI3T3	NC_020565.1	469823627	32,184		No
No	SAP052A	GQ900412.1	260066114	32,445		No
No	SAP017A	GQ900382.1	260066084	32,650		No
No	pI6T6	NC_020567.1	472455813	32,700		No
No	pCM05	GQ900387.1	260066089	33,660		No
No	pWBG747	GQ900399.1	260066101	33,701		No
No	pWBG746	GQ900390.1	260066092	33,702		No
No	D139	GG730186.1	282317966	33,841		No
No	pSK64	GQ915268.1	260066073	34,784		No
No	pEDINA	NC_010077.1	161598491	34,986		No
No	SAP076A	GQ900427.1	260066129	35,114		No
No	pSK60	GQ915267.1	260066072	35,175		No
No	pSK74	GQ915266.1	260066071	35,327		No
No	pTZ2162	NC_010419.1	170780412	35,380		No
No	SAP078A	GQ900430.1	260066132	35,508		No
No	SAP077A	GQ900428.1	260066130	35,510		No
No	pSK79	GQ900489.1	260177115	35,850		No
No	SAP102A	GQ900496.1	260177122	36,082		No
No	pSK21	GQ900490.1	260177116	37,046		No
No	SAP075A	GQ900486.1	260177112	37,050		No
pSK41 type	pUSA03	NC_007792.1	87159847		37,136	pSK41 type
No	SAP067A	GQ900483.1	260177109	37,206		No
pSK41 type	pI5S5	NC_020535.1	469816294		37,285	pSK41 type
No	SAP054A	GQ900477.1	260177103	37,475		No
No	pWBG758	NC_013329.1	260667551	38,045		No
pWBG749 t	pWBG749	GQ900391.1	260066093		38,087	pWBG749 type
pWBG749 t	pWBG745	NC_013325.1	260667421		38,204	pWBG749 type
No	pETB	NC_003265.1	17227177	38,211		No
No	pSK53	GQ915270.1	260066075	38,886		No
pSK41 type	pV030-8,	NC_010279.1	166153441		39,041	pSK41 type
pWBG4 typ	pSA737	NC_020227.1	448819501		39,287	pWBG4 type
pWBG4 typ	pCH91	NC_021076.1	484868062		39,287	pWBG4 type
No	SAP057A	GQ900415.1	260066117	39,308		No
pSK41 type	pPR9,	NC_013653.1	281427745		41,715	pSK41 type
No	pSK23	GQ900491.1	260177117	41,993		No
pSK41 type	SAP069A,	GQ900422.1	260066124		42,198	pSK41 type
pSK41 type	SAP080A,	GQ900433.1	260066135		43,892	pSK41 type
pSK41 type	SAP082A,	GQ900434.1	260066136		44,116	pSK41 type
pWBG749 t	pWBG748	GQ915265.1	260066070		44,964	pWBG749 type
No	pSK156	GQ900448.1	260066150	45,052		No
pSK41 type	pSK41,	AF051917.1	3676412		46,445	pSK41 type
pSK41 type	SAP079A,	GQ900432.1	260066134		47,322	pSK41 type
pSK41 type	SAP014A,	GQ900379.1	260066081		50,429	pSK41 type
pSK41 type	SAP068A,	GQ900421.1	260066123		50,500	pSK41 type
pSK41 type	pGO1,	NC_012547.1	226464389		54,000	pSK41 type
No	pWBG762	GQ900475.1	260177101	54,023		No
pSK41 type	pLW043,	NC_005054.1	33416233		57,889	pSK41 type
No	pETBTY825	NC_022598.1	555229551	60,563		No
